# Nonalcoholic Fatty Liver Disease Is Associated With Low Skeletal Muscle Mass in Overweight/Obese Youths

**DOI:** 10.3389/fped.2020.00158

**Published:** 2020-04-15

**Authors:** Lucia Pacifico, Francesco Massimo Perla, Gianmarco Andreoli, Rosangela Grieco, Pasquale Pierimarchi, Claudio Chiesa

**Affiliations:** ^1^Department of Pediatrics, Sapienza University of Rome, Rome, Italy; ^2^Institute of Translational Pharmacology, National Research Council, Rome, Italy

**Keywords:** NAFLD, NASH, youths, muscle mass, dual-energy X-ray absorptiometry

## Abstract

**Background:** Recent studies in adult non-elderly and elderly individuals have reported a link between nonalcoholic fatty liver disease (NAFLD) and sarcopenia. Nonetheless, whether this relationship would be found outside these populations it is still unknown. Hence, we evaluated the relationship between NAFLD and skeletal muscle mass in children and adolescents with overweight/obesity.

**Methods:** Two-hundred and thirty-four overweight/obese youths were enrolled. NAFLD was diagnosed by ultrasononography, after exclusion of infectious and metabolic disorders. Forty of the patients with NAFLD had also liver biopsy. Total and regional lean body mass and total fat mass measurements were obtained by dual-energy X-ray absorptiometry. The relative muscle mass (RMM) was defined as the percent of muscle mass (kg) relative to the sum of muscle and fat (kg) mass. Appendicular skeletal muscle mass (ASM) was calculated by the sum of muscle masses of the four limbs (kg), and expressed as percent of body weight.

**Results:** Subjects were stratified according to tertiles of RMM. The prevalence of abdominal obesity, dyslipidemia, insulin resistance, metabolic syndrome, NAFLD as well as biopsy-proven nonalcoholic steatohepatitis (NASH) was significantly increased in the lowest tertile of RMM. After controlling for age, sex and Tanner stage, children in the lowest tertile of RMM had an increased risk for NAFLD (OR= 2.80, 95% CI=1.57–5.02) compared to those in the other two tertiles. This association persisted after additional adjustments for clinical and metabolic variables. Similarly, the risk of NAFLD in the lowest tertile of ASM/weight index was significantly higher compared to those in the other two tertiles after adjustment for the above confounders.

**Conclusions:** This is the first study to establish an independent association between low muscle mass and NAFLD/NASH in overweight/obese youths. Considering the worldwide increase of pediatric obesity, measurements of muscle mass may serve as useful method of identifying among obese children those at high metabolic risk who may need intensive lifestyle interventions to prevent NAFLD and its progression.

## Introduction

With the worldwide epidemic of obesity, nonalcoholic fatty liver disease (NAFLD) has emerged as the most prevalent chronic liver disease in adults as well as youths (1), and a rising indication for liver transplantation. NAFLD include a broad range of liver damage from simple steatosis, nonalcoholic steatohepatitis (NASH), to cirrhosis (2). Both adult and pediatric patients with NAFLD often manifest features of metabolic syndrome (MetS) (e.g., abdominal obesity, increased blood pressure, atherogenic dyslipidemia, insulin resistance, and glucose abnormalities), and thus are at greater risk for cardiovascular disease (CVD) (3–5). Although significant progresses in our knowledge of the pathophysiology of NAFLD have been achieved, mechanisms accounting for excess fat in the liver have not yet fully clarified. Therefore, the identification of all major factors affecting development of NAFLD earlier in life is crucial to prevent the progression of liver damage.

Recently, emerging evidence suggests that reduced skeletal muscle mass contributes to the risk of many chronic diseases including chronic liver diseases (6). The loss in lean body mass, that is sarcopenia, has long been associated with liver cirrhosis (7) as well as with a poor prognosis in patients with end-stage liver disease (8). According to the revised European consensus on definition and diagnosis of sarcopenia (9), this disease is characterized by low muscle quantity and quality. Infiltration of fat within and around skeletal muscle, that is myosteatosis, is directly related to age and adiposity, with an increased risk of adverse outcomes (10, 11). Although sarcopenia has long been thought to be a disease of the elderly (9), it has been recently reported to occur earlier in patients with cardiometabolic disorders such as obesity, diabetes mellitus, MetS and CVD (12). Indeed, several studies have reported in the adult non-elderly and elderly populations a link between sarcopenia and NAFLD (13), highlighting sarcopenia as an emerging risk factor for NAFLD and its progression. Nonetheless, whether this relationship would be found outside these populations it is still unknown. We here report the results of an observational, cross-sectional study investigating the relationship between NAFLD and skeletal muscle mass in a pediatric population with overweight/obesity.

## Methods

### Patients

We enrolled 234 children and adolescents with overweight/obesity [body mass index (BMI) > 85th percentile according to age- and gender-specific percentiles of BMI] at the outpatient Clinics of the Department of Pediatrics, Sapienza University of Rome. Subjects were included if they were aged 6-18 years, nondiabetic, free from chronic diseases (including kidney, endocrinologic, and liver disorders) as well as from conditions known to influence body composition.

The study was approved by the Policlinico Umberto I Hospital Ethical Committee, and the parents of all participants gave informed consent.

### Clinical and Laboratory Data

Anthropometric measurements were obtained with standard methods. Weight and Height were determined using an electronic scale and a wall-mounted stadiometer, respectively. BMI was calculated as body weight in kg divided by the height in meters squared. The pubertal status was evaluated by the Tanner stage. The degree of obesity was quantified using Cole's least mean-square method, which expresses BMI as standard deviation score (SDS) (14).

After the subject fasted overnight, blood samples were collected for the determination of glucose, insulin, total cholesterol, high-density lipoprotein cholesterol (HDL-C), triglycerides (TG), alanine aminotransferase (ALT), and aspartate aminotransferase. Insulin resistance was assessed by the homeostasis model assessment of insulin resistance (HOMA-IR) (15).

### Ultrasound Examination of the Liver

Liver ultrasonography was performed by a single operator blinded to clinical and laboratory data. Diagnosis of fatty liver was based upon liver echogenicity exceeding that of the renal cortex and spleen, attenuation of ultrasound wave, loss of definition of the diaphragm, and poor delineation of the intrahepatic architecture (16).

### Dual Energy X-ray Absorptiometry Scans

Total and regional lean body mass (kg) and total fat mass (kg) were obtained by a total body scanner (Hologic QDR-4500W). The relative muscle mass (RMM) was defined as the percent of muscle mass relative to the sum of muscle and fat mass [e.g., 100 x muscle mass (kg) / muscle mass (kg) + fat mass (kg)], a measure to estimate the contribution of relative muscle mass to body composition (17–19). Appendicular skeletal muscle mass (ASM) was calculated by the sum of muscle masses in the four limbs (kg), and expressed as percent of body weight [ASM/weight (kg) x 100]. We also calculated ASM after adjusting for height squared (ASM/ht^2^) (17–19).

### Liver Biopsy

A subgroup of obese children with ultrasound-diagnosed NAFLD had also liver biopsy to assess either the presence of NASH, or other competing liver diseases. Percutaneous needle liver biopsy was performed as previously described (15). The histologic features of steatosis, lobular and portal inflammation, and hepatocyte ballooning, and fibrosis were scored according to the NAFLD Clinical Research Network criteria (20). Diagnosis of NASH was based on the presence of steatosis with necroinflammation and hepatocyte ballooning (21).

### Definitions

NAFLD was defined as the presence of fat in the liver on ultrasound (US) in the absence of an alternate identifiable cause. High blood pressure (BP) was defined by BP ≥ 95th percentile for age, sex, and height (22). High waist circumference (WC), high TG, and low HDL-C were defined using age- and gender-specific percentiles (23). Impaired fasting glucose was defined by a value of fasting glucose ≥ 5.6 mmol/L. MetS diagnosis was based on the presence of at least 3 risk factors: high WC, elevated BP, low HDL-C levels, hypertriglyceridemia and glucose impairment. Insulin resistance was established on the basis of the 90^th^ percentile of HOMA-IR specific for age and gender in overweight/obese children (24).

### Statistical Analysis

Data are expressed as n (%), mean (SD), or median (interquartile range). Overweight/obese children, with and without NAFLD, were stratified into tertiles of the total skeletal muscle mass. Differences among groups in quantitative variables were evaluated by one-way analysis of variance or Kruskal–Wallis test, as appropriate. Proportions were compared by the chi-square test. Partial correlation and linear regression coefficients were used to evaluate the relationship between variables. In order to assess the risk of NAFLD in the first (lowest) tertile of RMM as well as in the first (lowest) tertile of ASM/weight index compared to the combined second and third tertiles, we performed multiple logistic regression analysis controlled for age, sex, Tanner stage, and clinical confounders. Since the prevalence of cardiometabolic risk factors in the first tertile was markedly different to that observed in the second and third tertiles, the latter two tertiles have been combined.

## Results

### Characteristics of Study Population

Descriptive characteristics of participant samples according to tertiles of RMM are summarized in Table 1. The subjects in the lowest tertile of RMM had the greatest BMI, BMI-SDS, WC, total body fat mass as well as the highest TG/HDL-C ratio, insulin, and HOMA-IR values compared with those in the other two tertiles of RMM. In comparison with subjects in the lowest RMM tertile, those in the middle or highest RMM tertile were more likely to be taller, and to have greater total lean body mass, absolute ASM, ASM/weight index, and ASM/ht^2^. There was a near-significance difference in age across the tertiles of RMM. Moreover, reduced RMM was significantly associated with an increased prevalence of NAFLD (*P* = 0.006). Conversely, there were no significant differences in Tanner stage, BP, total cholesterol, liver enzymes and glucose.

**Table 1:** Characteristics of study population according to tertiles of RMM^a^.

	**RMM**			***P***
	**Tertile1**	**Tertile II**	**Tertile III**	
Number of subjects	78	75	81	
Age, years	11.3 (2.3)	11.5 (2.8)	12.5 (3.0)	0.06
Male sex, *n* (%)	38 (48.7)	33 (44.0)	61 (75.3)	0.001
Prepubertal status, *n* (%)	15 (19.2)	16 (21.3)	14 (17.2)	0.26
Weight, kg	65.0 (21.0)	59.3 (22.1)	64.1 (21.1)	0.21
Height, cm	149.7 (14.4)	151.7 (17.4)	158.9 (18.0)	0.01
BMI (kg/m^2^)	28.3 (4.5)	24.7 (3.7)	24.6 (3.3)	<0.0001
BMI-SD score	2.13 (0.40)	1.70 (0.39)	1.60 (0.39)	<0.0001
Waist circumference, cm	91.2 (13.2)	84.8 (14.0)	84.6 (12.0)	0.002
Systolic BP, mmHg	111 (11)	111 (9)	112 (13)	0.81
Diastolic BP, mmHg	69 (9)	68 (8)	69 (9)	0.82
Total cholesterol, mg/dL	170 (39)	177 (50)	163 (41)	0.19
HDL-C, mg/dL	46 (13)	47 (12)	49 (10)	0.17
Triglycerides, mg/dL	97(72-141)	91 (65-128)	76 (52–123)	0.031
TG/HDL-C ratio	2.1 (1.3-3.6)	1.9 (1.2-3.3)	1.6 (0.9-2.8)	0.029
AST, U/L	25 (20–34)	25 (20–30)	23 (19–29)	0.22
ALT, U/L	25 (17–47)	22 (15–35)	20 (15–32)	0.15
Glucose, mg/dL	4.8 (0.8)	4.8 (0.45)	4.8 (0.39)	0.84
Insulin, μU/mL	18 (12–24)	14 (9–19)	13 (9–18)	0.004
HOMA-IR	3.7 (2.5-4.8)	2.8 (1.9-3.9)	2.8 (2.0-4.0)	0.015
Total body fat mass, kg	28.0 (10.0)	22.8 (9.0)	19.0 (5.9)	<0.0001
Total lean body mass, kg	31.4 (9.6)	32.5 (12.7)	39.6 (14.6)	<0.0001
RMM, %	53.0 (3.3)	58.9 (1.5)	66.9 (4.8)	<0.0001
ASM, kg	15.1 (4.36)	15.5 (7.0)	20.7 (7.94)	<0.0001
ASM/weight index, %	24.5 (1.73)	26.2 (2.86)	30.8 (7.42)	<0.0001
ASM/ht^2^	6.7 (1.0)	6.4 (1.6)	7.6 (2.0)	0.001
NAFLD, *n* (%)	43 (55.2)	25 (33.3)	27 (33.3)	0.006

*RMM, relative muscle mass; BMI, body mass index; BMI-SDS, BMI-SD score; BP, blood pressure; TG, triglycerides; HDL-C, high-density lipoprotein cholesterol; AST, aspartate aminotransferase; ALT, alanine aminotransferase; HOMA-IR, homeostasis model assessment of insulin resistance; ASM, appendicular skeletal muscle mass*.

a *Tertile I, RMM: < 56.72; tertile II, RMM: 56.72–61.99; tertile III, RMM: > 61.99*.

*Results are expressed as n (%), mean (SD) or median (interquartile range)*.

### Relationship Between RMM and Cardiometabolic Risk Factors

In all study children and adolescents, after controlling for age, sex, and Tanner stage, RMM were negatively correlated with WC, diastolic BP, ALT, TG, TG/HDL ratio, insulin, and HOMA-IR values (Table 2). When the analysis was limited to patients with NAFLD, RMM were significantly associated with WC, systolic BP, TG, HDL-C and TG/HDL-C ratio, ALT, insulin and HOMA-IR values. In the subjects without NAFLD, RMM was significantly correlated only with WC [B coefficient,−0.311 (95% CI, -0.419/-0.204); *P* < 0.0001].

**Table 2:** Age-, gender, and pubertal status- adjusted linear regression coefficients between RMM and clinical variables.

	**All cases**	**NAFLD**
	**B coefficients (95% CI)**	**B coefficients (95% CI)**
Waist circumference, cm	−0.320 (-0.387,−0.253)^§^	−0.327 (-0.414,−0.240)^§^
Systolic BP, mmHg	−0.081 (-0.167, 0.006)	−0.175 (-0.290,−0.060)^*^
Diastolic BP, mmHg	−0.101 (-0.199,−0.002)^*^	−0.107 (-0.261, 0.047)
ALT	−0.035 (-0.060,−0.009)^+^	−0.032 (-0.061,−0.002)^*^
HDL-C, mg/dL	0.057 (-0.13, 0.127)	0.173 (0.07, 0.278)^+^
Triglycerides, mg/dL	−0.014 (-0.25,−0.002)^*^	−0.015 (-0.029,−0.001)^*^
TG/HDL-C ratio	−0.454 (-0.78,−0.12)^+^	−0.500 (-0.87,−0.13)^+^
Glucose	−0.307 (-1.76, 1.15)	−0.903 (-2.54, 0.74)
Insulin	−0.136 (-0.204,−0.067)^§^	−0.125 (-0.197,−0.052)^+^
HOMA-IR	−0.396 (-0.632,−0.161)^+^	−0.346 (-0.585,−0.108)^+^

*RMM, relative muscle mass; NAFLD, nonalcoholic fatty liver disease; CI, confidence interval; BP, blood pressure; ALT, alanine aminotransferase; TG, triglycerides; HDL-C, high-density lipoprotein cholesterol; HOMA-IR, homeostasis model assessment of insulin resistance*.

* *P < 0.05*;

+ *P < 0.01*;

§ *P < 0.0001*.

### Cardiometabolic Profile Across RMM Tertiles in the Study Population

The prevalence of high WC, high TG, low HDL-C, insulin resistance, NAFLD and MetS was significantly increased in the lowest tertile of RMM (Table 3).

**Table 3:** Prevalence of metabolic syndrome and its individual components according to RMM tertiles among the study population.

	**RMM**			
	**Tertile I (*n* = 78)**	**Tertile II (*n* = 75)**	**Tertile III (*n* = 81)**	***P* for linear trend**
Central obesity, % (95% CI)	62.8 (52.1-73.5)	34.6 (23.8-45.4)	13.6 (6.1-21.1)	<0.0001
Elevated BP, % (95% CI)	29.5 (19.4-39.6)	17.3 (8.7-25.9)	27.1 (17.4-36.8)	0.75
High TG, % (95% CI)	35.9 (25.3-46.5)	21.3 (12.0-30.6)	21.0 (12.1-29.9)	0.033
Low HDL-C, % (95% CI)	38.5 (27.7-49.3)	20.0 (11.0-29.0)	19.7 (11.0-28.4)	0.008
Glucose ≥ 5.6 mmol/L, % (95% CI)	1.3 (0.2-6.9)^*^	4.0 (1.4-11.1)^*^	3.7 (1.27-10.3)^*^	0.36
Insulin resistance, % (95% CI)	70.5 (60.4-80.6)	52.0 (35.0-69.0)	51.8 (40.9-62.7)	0.023
NAFLD, % (95% CI)	55.2 (32.9-70.5)	33.3 (22.6-44.0)	33.3 (23.1-43.5)	0.006
Metabolic syndrome, % (95% CI)	29.5 (19.3-39.7)	12.0 (4.6-19.3)	3.7 (1.3-10.3)^*^	<0.0001

*RMM, relative muscle mass; CI, confidence interval; BP, blood pressure; TG, triglycerides; HDL-C, high-density lipoprotein cholesterol; NAFLD, nonalcoholic fatty liver disease*.

* *For these percentages, the exact 95% CI was calculated using the Wilson method*.

To evaluate the potential independent contribution of RMM on NAFLD, multiple logistic regression analyses were performed (Table 4A). After controlling for age, sex and Tanner stage, children in the lowest tertile of RMM had an increased risk for NAFLD (OR = 2.80, 95% CI =1.57-5.02) compared to those in the other two tertiles. This association persisted after adjusting for potential confounders such as central obesity, elevated BP, elevated TG, low HDL-C, and insulin resistance, although the strength of association was slightly attenuated. When MetS (as a single clinical entity) was entered in the regression model in addition to age, gender and Tanner stage, the association remained statistically significant (OR = 2.20, 95% CI = 1.19-4.05).

**Table 4A:** Adjusted odds ratio (95% CI) of the lowest tertile of RMM for NAFLD.

	**RMM**		
	**I**	**II and III**	***P* value**
Adjusted model 1	2.80 (1.57-5.02)	1.00 (referent)	0.001
Adjusted model 2	2.18 (1.17-4.07)	1.00 (referent)	0.014
Adjusted model 3	2.18 (1.13-4.18)	1.00 (referent)	0.019
Adjusted model 4	2.20 (1.19-4.05)	1.00 (referent)	0.012

We also evaluated the adjusted associations of ASM/weight index with NAFLD (Table 4B). In model 1, controlled for age, sex and Tanner stage, the risk of NAFLD (OR = 2.99, 95% CI =1.41-6.31) in the lowest tertile of ASM/weight index was significantly higher compared to that in the other two tertiles. These results remained unchanged after additional adjustments (including central obesity, elevated BP, elevated TG, low HDL-C, and insulin resistance, or MetS).

**Table 4B:** Adjusted odds ratio (95% CI) of the lowest tertile of ASM/weight index for NAFLD.

	**ASM**		
	**I**	**II and III**	***P* value**
Adjusted model 1	2.99 (1.41-6.31)	1.00 (referent)	0.004
Adjusted model 2	2.29 (1.04-5.06)	1.00 (referent)	0.04
Adjusted model 3	2.33 (1.01-5.40)	1.00 (referent)	0.048
Adjusted model 4	2.54 (1.16-5.58)	1.00 (referent)	0.02

*Model 1: adjusted for age, gender and pubertal status; Model 2: adjusted for age, gender, pubertal status, and central obesity; Model 3: adjusted for age, gender, pubertal status, central obesity, high blood pressure, elevated triglycerides, low high-density lipoprotein cholesterol, and insulin resistance; Model 4: adjusted for age, gender, pubertal status and MetS*.

*CI, confidence interval*.

### Findings in Children With Biopsy-Proven NAFLD

Twenty-four children (60.0%) had definite-NASH, while 16 (40.0%) had not-NASH. In comparison with children and adolescents with not-NASH, those with NASH showed significantly lower RMM [mean, 55.7 (SD, 6.0) vs. 63.4 (6.0) %; *P* < 0.0001] and lower ASM/weight index [mean, 25.6 (SD, 2.8) vs. 28.6 (2.9) %; *P* = 0.006]. Also, the prevalence of NASH was significantly increased in the lowest tertile of RMM [70.8 (95% CI, 61.8-79.8) % vs. 29.2 (20.2-38.2) %; *P* < 0.001] as well as in the lowest tertile of ASM/weight index [62.5 (95% CI, 47.5-77.5) % vs. 37.5 (22.5-52.5) %; *P* < 0.003] compared to those in the other two tertiles, respectively.

Some degree of fibrosis was present in 72.5% of patients with histologically diagnosed NAFLD, of whom 30% showed stage 1, 40% stage 2 and 2.5% stage 3 fibrosis. There were no significant differences in RMM and ASM/weight index between patients with fibrosis grade ≥2 and those with fibrosis ≤ 1.

## Discussion

To our knowledge, this is the first study to assess in a pediatric population the relationship of skeletal muscle mass with NAFLD. We demonstrated that (1) overweight/obese youths with lower muscle mass have a greater risk of NAFLD compared to those with higher muscle mass; (2) the inverse association between NAFLD and muscle mass in children and adolescents is independent from anthropometric and metabolic variables; and (3) overweight/obese youths with lower muscle mass have a greater prevalence of cardiometabolic risk factors (e.g., central obesity, dyslipidemia, and insulin resistance) as well as MetS. In the subgroup of obese patients with US-diagnosed NAFLD who underwent liver biopsy, we also demonstrated the association between reduced muscle mass and NASH.

Several cross-sectional studies in adult patients have showed that sarcopenia is associated with NAFLD, NASH and NAFLD-associated advanced fibrosis (25–29). Notably, in a nationally representative sample of both obese and non-obese Korean adult individuals, Lee et al. (25) provided robust evidence of an independent association of NAFLD with sarcopenia. They demonstrated that patients with sarcopenia had a higher risk of NAFLD independently of the status of obesity as well as of MetS compared with subjects with a preserved muscle mass. More recently, in a large, longitudinal population-based cohort study, Kim et al. confirmed this association and suggested a causal link (30). The authors tested the effects of RMM modifications over time on the occurrence of new NAFLD or the resolution of pre-existing NAFLD (30). Skeletal muscle mass index (SMI) as assessed at baseline was inversely related to incident NAFLD and positively related to the resolution of pre-existing NAFLD. Furthermore, an increase in SMI over 1-year period had significant favorable effects either on the development of new NAFLD or the improvement of pre-existing NAFLD, even after controlling for glycometabolic variables and baseline SMI (30).

Although in 1984 Forbes (31) described for the first time a low muscle mass phenotype in obese children, since then very little attention has been paid to its metabolic implications in pediatrics. Only recently, children and adolescents with low muscle mass and strength have been shown to be at increased risk of developing metabolic dysfunction and CVD (18, 32–35), as previously reported in the adult nonelderly and elderly populations (6). In a large sample of U.S. youth, aged 8–20 years, Kim et al. (18) demonstrated an inverse association between RMM and cardiometabolic risk factors. In addition, they showed that the odds of having an adverse level for all risk factors with the exception of diastolic BP gradually diminish as RMM increases. A more recent study involving 660 apparently healthy adolescents, showed that those with low muscle mass (into the first quartile) had higher cardiometabolic risk (higher values of BMI z-score, WC, systolic BP, diastolic BP, TG, TC/HDL-C, insulin, HOMA-IR, and MetS z-score) than adolescents in the other quartiles regardless of nutritional status (17). Furthermore, low muscle mass increased the obesity-related cardiometabolic risk. In the present study involving a Caucasian pediatric population with overweight/obesity, we confirmed the inverse relationship between low muscle mass and cardiometabolic risk factors, and we first showed an independent association between low muscle mass (total and appendicular) and NAFLD as well as NASH.

There is growing recognition that reduced lean tissue, notably skeletal muscle, can co-occur in the presence of obesity, the so-called sarcopenic obesity (12, 36–38). Clinical evaluation of muscle mass in the obese subjects, however, is a real pitfall, since a reduced skeletal muscle mass may be masked by the presence of excess fat (37). The concordance of these two conditions, e.g., low level of muscle mass and excess weight, is associated with worse clinical outcomes than is either condition alone (39–41); therefore, attempts to preserve muscle mass and to lessen the consequences of low muscle mass might be more fruitful if initiated at childhood than if initiated at adulthood. Accordingly, in obese children a systematic muscle mass assessment is needed to improve diagnosis and treatment of those presenting a disparity in muscle and fat stores at an early stage. As such, more and more attention is being paid to the reference technology as well as to the diagnostic criteria to assess muscle mass in different contexts and populations including obese children.

Currently, various techniques are available for estimating or measuring muscle mass. Unfortunately, no consensus has yet been reached on the best technique to estimate or measure it (42). Among them, computed tomography (CT) and magnetic resonance imaging (MRI) are ideal in terms of accuracy, but their routine use in many clinical settings is compromized by the high cost of instrumentation, concerns of radiation exposure (for CT), contraindications for scanning (for MRI), and limited access to equipment (36–38, 42). Thus, both techniques are not suitable for population screening, nor available at early asymptomatic stages of the disease (37). Unsurprisingly, use of these gold standard techniques to assess the muscle mass in the clinical pediatric setting has been limited to children with end-stage organ failure and/or increased fat stores requiring imaging evaluation of the underlying chronic diseases while awaiting solid organ transplantation (43, 44).

A yet poorly explored element of sarcopenia has been quantification of fatty infiltration of muscle. However, evaluation of fat deposition in muscles is complex, far from clinical practice, especially in children and adolescents, and may be obtained only using imaging techniques or more invasive methods like muscle biopsy (45). CT and MRI are considered the gold standard for intramuscular and intermuscular fat evaluation, respectively, while other body composition methods including DEXA do not allow to evaluate muscle quality parameters. Accordingly, this limitation warrants further studies assessing in a selected group of pediatric patients the relevance of myosteatosis by imaging techniques in childhood “sarcopenic obesity.”

Bearing all these considerations in mind, despite many limitations, DEXA has been proposed as the standard technique for assessing muscle mass and body composition in research and clinical practice because of its ease of use, relatively low cost, minimal radiation exposure, short scan time and accessibility (42).

In addition to the commonly reported appendicular lean-mass estimates (i.e., estimation of the muscle masses in the four limbs, representing about 75% of total body muscle mass) (37, 42), using DEXA we also measured the contribution of RMM to body composition. To this end, we followed the method proposed by Kim et al. (18) as variation of the measure initially introduced by Park et al. (46) to evaluate in adults the relationship between muscle mass and MetS. In children the muscle-to-fat ratio (MFR) has been established as the key indicator of low muscle mass with the potential to stratify the risk of complications (19, 47). Indeed, in obese youths, the presence of a low muscle mass in a combination with a high fat mass, which would result in low MRF, may act synergistically leading to a more severe cardiometabolic risk (19).

NAFLD diagnosis was based in the majority of participants on US examination after exclusion of infectious and metabolic disorders, while only a small sample size of children underwent liver biopsy, which is the current standard to define the presence and severity of NAFLD. Although US is the imaging modality most widely used for the noninvasive assessment of liver steatosis, due to its significant advantages such as being largely available, relatively non-expensive, and easy to use, it has low sensitivity, particularly in children who have lower degrees of steatosis (e.g., involving <33% of hepatocytes). In addition, US is inaccurate for quantification of steatosis in youths. For these reasons, the recent guidelines from the NASPGHAN Expert Committee have recommended ALT as the best screening test for NAFLD in children (48). However, in a very recent study, two screening strategies were compared: the NASPGHAN strategy using an ALT cut-off of >2x the gender-specific upper limit of normal and the ESPGHAN strategy using elevated ALT >45 IU/L and/or fatty liver on ultrasound. The study showed that by relying on ALT values alone to screen for NAFLD, suspected NAFLD might be missed in many children who are at risk to develop the disease (49). In our study, it is possible that some subjects with US-diagnosis of NAFLD (due to low sensitivity) were enrolled in the control groups. However, the possible inclusion of controls with NAFLD may have led to underestimation of the differences in the RMM between cases and controls rather than the opposite.

Although this study was not designed to clarify the pathogenic link between sarcopenia and NAFLD, we acknowledge that the two conditions have in common several pathophysiologic processes, especially insulin resistance, chronic inflammation, and decreased physical activity (50). Skeletal muscle is the most effective organ for whole-body insulin-mediated glucose disposal (51), and therefore is the key element for maintaining effective glucose homeostasis in many chronic diseases. As such, the loss of muscle mass reduces the quantity of the primary target for insulin, favoring glucose intolerance and gluconeogenesis, which are key to the pathogenesis of NAFLD. Interestingly, Petersen et al. (52) demonstrated that, after high carbohydrate meals, young insulin-resistant subjects showed a marked defect in muscle glycogen synthesis and diverted much more of their ingested energy into hepatic *de novo* lipogenesis, resulting in increased hepatic triglycerides synthesis, while young insulin-sensitive individuals stored more of their ingested energy in liver and muscle glycogen. In our study, low muscle mass was related to NAFLD independently of insulin resistance. Thus, it is plausible that other mechanisms could be involved, including subclinical inflammation and enhanced oxidative stress. Subjects with sarcopenia have elevated concentrations of C-reactive protein and inflammatory cytokines, which may promote skeletal muscle catabolism (28, 53). Low-grade inflammation and oxidative stress may also have a relevant role for the occurrence of NAFLD and its progression. Moreover, skeletal muscle is regarded as an endocrine organ able to secrete a series of cytokines so-called myokines, which constitute a broad network among metabolic tissues and organs including the liver (54). Myokines produced and released by contracting skeletal muscles counteract systemic inflammation and modulate glucose and lipid metabolism (55). This might account for the well-known favorable effects of physical activity toward the diseases associated with “the diseasome of physical inactivity” (55). Importantly, there is a growing body of evidence supporting the beneficial effects of exercise and of physical fitness *per se* in the pathogenesis of NAFLD (56). As a matter of fact, exercise-only interventions (e.g., without modification of the diet) have been shown to result in a significant reduction of the amount of intrahepatic fat, even in the absence of significant weight changes (57). The influence of physical inactivity on both obesity and muscle reduction in children is common sense. Both physical inactivity and sedentary behavior with consequent muscle disuse can lead to a substantial decrease in lean body mass, creating a vicious cycle causing both progressive inactivity and sarcopenia.

Nutrition represents a key factor in the prevention and treatment of both sarcopenia and obesity. Sarcopenia is associated with an inadequate nutritional intake, and nutritional interventions are essential to improve sarcopenia and the subsequent morbidity and mortality in chronic liver diseases (58, 59). In contrast, obesity is a result of an excess consumption of energy, resulting in an imbalance between the energy intake and energy expenditure. As such, nutritional strategies for sarcopenic obesity should target not only an optimal energy intake in order to decrease excess fat mass, but also an optimal nutrient intake in order to increase skeletal muscle mass (60). Recent studies have shown that a combination of a moderate weight loss diet with concurrent exercise (especially resistance exercise) may improve body composition and physical performance in subjects with sarcopenic obesity (61).

Gut dysbiosis might have a role in the development and progression of NAFLD; therefore, the manipulation of gut microbiota with probiotics might prove an effective treatment strategy, particularly in subjects who are noncompliant to lifestyle interventions (62). Few studies in children have yielded promising results; however, several aspects of probiotic beneficial action in NAFLD (e.g., type of strain and doses) still need further elucidation (62). Of note, recently researchers are focusing their interests on the possible involvement of gut microbiota in the pathophysiology of sarcopenia and physical frailty (63). Alterations in the gut microbiota composition could in fact promote chronic inflammation and anabolic resistance, ultimately conditioning reduced muscle mass, impaired muscle function and adverse clinical outcomes (63). Thus, the relationship between gut microbiota and sarcopenic obesity remains a very promising area of research for the future.

Some limitations of the present study should be considered: (a) the cross-sectional nature, that does not allow determination of cause-and-effect relationship; (b) the relatively small sample size of children with biopsy-confirmed diagnosis of NAFLD; (c) missing data on physical activity; and (d) lack of data on modifications in muscle strength and quality.

Nevertheless, despite these limitations, this is the first study to establish a close and independent association between low muscle mass and NAFLD/NASH in overweight/obese youths. Considering the worldwide increase of pediatric obesity, measurements of muscle mass may serve as useful method of identifying among obese children those at high cardiometabolic risk who may need more detailed medical examinations, and lifestyle interventions including adequate nutritional support and intensive exercise prescriptions for prevention of NAFLD and its progression.

## Data Availability Statement

The datasets generated for this study are available on request to the corresponding author.

## Ethics Statement

The studies involving human participants were reviewed and approved by Policlinico Umberto I Hospital Ethics Committee, Sapienza University. Written informed consent to participate in this study was provided by the participants' legal guardian/next of kin.

## Author Contributions

LP, FP, and CC conceptualized and designed the study. LP, FP, and RG collected the data. LP, PP, and CC analyzed and interpreted the data. LP and CC wrote the manuscript and all authors participated to the discussion of results and critically commented the manuscript for the final approval.

### Conflict of Interest

The authors declare that the research was conducted in the absence of any commercial or financial relationships that could be construed as a potential conflict of interest.
